# Retraction Note: Autism Tsunami: The Impact of Rising Prevalence on the Societal Cost of Autism in the United States

**DOI:** 10.1007/s10803-023-06016-4

**Published:** 2023-06-05

**Authors:** Mark Blaxill, Toby Rogers, Cynthia Nevison

**Affiliations:** 1 Holland Center, Minnetonka, MN USA; 2 Pasadena, CA USA; 3 Institute for Alpine and Arctic Research, University of Colorado, Boulder, CO USA

**Retraction Note: Journal of Autism and Developmental Disorders (2021) 52:2627–2643**

10.1007/s10803-021-05120-7

The Publisher and the Editors-in-Chief are retracting this article. After publication concerns were raised with respect to the methodology used in this article. Post-publication review by a number of experts in the field has confirmed the following:

- Misrepresentation of the incidence of autism and insufficient attention given to various other factors that might account for apparent changes in rates
- Use of unrepresentative data
- No valid justification for the mechanisms proposed for prevention
- Arbitrary and insufficiently justified use of the birth cohort 1931 as an origin for the time trend
- Use of higher estimates and assumptions that inflated costs, together with failure to consider discrete events that occurred in referent years, including changes in definitions, alternative surveillance methods, and alternative explanations which may have contributed to changes in diagnosis.

In addition, the Conflict of Interest statement does not accurately reflect the non-financial interests of the authors.

The Editors-in Chief therefore no longer has confidence in the conclusions presented. Mark Blaxill, Toby Rogers and Cynthia Nevison disagree with this retraction.

